# cMRI-BED: A novel informatics framework for cardiac MRI biomarker extraction and discovery applied to pediatric cardiomyopathy classification

**DOI:** 10.1186/1475-925X-14-S2-S7

**Published:** 2015-08-13

**Authors:** Vanathi Gopalakrishnan, Prahlad G Menon, Shobhit Madan

**Affiliations:** 1 Department of Biomedical Informatics, University of Pittsburgh, Pittsburgh, PA, USA; 2 Intelligent Systems Program, University of Pittsburgh, Pittsburgh, PA, USA; 3 Department of Computational and Systems Biology, University of Pittsburgh, Pittsburgh, PA, USA; 4 Department of Bioengineering, University of Pittsburgh, Pittsburgh, PA, USA; 5 Department of Radiology, University of Pittsburgh, Pittsburgh, PA, USA; 6 Pediatric Imaging Research Center, Children's Hospital of Pittsburgh, University of Pittsburgh Medical Center, Pittsburgh, PA, USA

## Abstract

**Background:**

Pediatric cardiomyopathies are a rare, yet heterogeneous group of pathologies of the myocardium that are routinely examined clinically using Cardiovascular Magnetic Resonance Imaging (cMRI). This gold standard powerful non-invasive tool yields high resolution temporal images that characterize myocardial tissue. The complexities associated with the annotation of images and extraction of markers, necessitate the development of efficient workflows to acquire, manage and transform this data into actionable knowledge for patient care to reduce mortality and morbidity.

**Methods:**

We develop and test a novel informatics framework called cMRI-BED for biomarker extraction and discovery from such complex pediatric cMRI data that includes the use of a suite of tools for image processing, marker extraction and predictive modeling. We applied our workflow to obtain and analyze a dataset of 83 de-identified cases and controls containing cMRI-derived biomarkers for classifying positive versus negative findings of cardiomyopathy in children. Bayesian rule learning (BRL) methods were applied to derive understandable models in the form of propositional rules with posterior probabilities pertaining to their validity. Popular machine learning methods in the WEKA data mining toolkit were applied using default parameters to assess cross-validation performance of this dataset using accuracy and percentage area under ROC curve (AUC) measures.

**Results:**

The best 10-fold cross validation predictive performance obtained on this cMRI-derived biomarker dataset was 80.72% accuracy and 79.6% AUC by a BRL decision tree model, which is promising from this type of rare data. Moreover, we were able to verify that mycocardial delayed enhancement (MDE) status, which is known to be an important qualitative factor in the classification of cardiomyopathies, is picked up by our rule models as an important variable for prediction.

**Conclusions:**

Preliminary results show the feasibility of our framework for processing such data while also yielding actionable predictive classification rules that can augment knowledge conveyed in cardiac radiology outcome reports. Interactions between MDE status and other cMRI parameters that are depicted in our rules warrant further investigation and validation. Predictive rules learned from cMRI data to classify positive and negative findings of cardiomyopathy can enhance scientific understanding of the underlying interactions among imaging-derived parameters.

## Background

Cardiovascular Magnetic Resonance Imaging (cMRI) is currently regarded as the gold standard for the non-invasive acquisition and processing of high-resolution temporal images for myocardial function and tissue characterization [1]. cMRI is a diagnostic imaging modality with no ionizing radiation and is available in specialized care clinical centers where it is routinely used to discover sources of abnormalities in cardiac structure, function and dynamics. Its applicability to the detection and diagnosis of cardiomyopathies, particularly in pediatric populations, is of immense significance for timely detection, accurate disease sub-classification and effective clinical management options with follow-up care by the primary physician in consultation with cardiac radiologists and specialists. The large amounts of cMRI data acquired per patient leads to several complexities associated with the annotation of images and extraction of markers to differentiate the various subtle and rare forms of cardiomyopathies. These complexities necessitate the development of efficient informatics workflows among cardiologists, radiologists and primary care physicians to acquire, manage and transform this data into actionable knowledge for patient care to reduce mortality and morbidity. Furthermore, there is a clear need to capture, analyse and understand retrospectively obtained imaging data and the derived parameters to enhance early detection and timely monitoring of such pediatric heart diseases to lessen morbidities in adulthood. Cardiomyopathies are believed to be a frequent cause of sudden cardiac arrest in the young.

Past work in this area has typically been in the image processing domain, wherein the effort has gone into imaging bio-marker extraction, segmentation of global and local regions of interest [2], extraction of quantitative metrics such as volume estimation, morphological, functional or flow-based features [3,4] and finally automated tools for multi-modal image registration. To the best of our knowledge, the clinical workflows associated with the cMRI data acquisition and processing have not been studied from a machine learning perspective to identify areas of inefficiencies wherein intelligent computational tools could be developed to aid pediatric radiologists and cardiologists in the accurate assessment of cardiomyopathies using a multitude of imaging biomarkers. In this paper, we develop and test a novel informatics framework called cMRI-BED (Cardiovascular Magnetic Resonance Imaging Biomarker Extraction and Discovery) that includes predictive modeling of retrospectively collected, de-identified cMRI and medical record data to extract classification rules that augment knowledge obtained from standard practice. We present our preliminary findings from the application of this workflow to a dataset containing positive and negative findings for a subset of pediatric patients evaluated for cardiomyopathies.

Cardiovascular disease is the #1 leading cause of death worldwide [5]. Cardiomyopathy (CM) generally refers to a rather rare, yet diverse group of diseases of the heart muscle that are classified according to anatomy and physiology into the following types: Hypertrophic cardiomyopathy (HCM), Dilated cardiomyopathy (DCM), Arrhythmogenic right ventricular cardiomyopathy/dysplasia (ARVC/D), Restrictive cardiomyopathy (RCM) and unclassified cardiomyopathies (NCM). In 1996, a highly cited scientific statement from the American Heart Association (AHA) proposed contemporary definitions and classification of primary and secondary cardiomyopathies that took into account molecular genetics in cardiology [6]. A recent article thoroughly illustrates the various types of common and rare cardiomyopathies, and their classification based on specific morphological and functional phenotypes [7].

cMRI is a popular non-invasive technology for cardiomyopathy evaluation. The basic protocols for cardiomyopathy assessment using cMRI are discussed and illustrated in [7]. A further discussion of assessment of rare cardiomyopathies using cMRI is presented in [8]. Standardized cMRI protocols are reviewed in [9]. cMRI has recently emerged as a powerful tool for detecting cardiovascular biomarkers [10]. It is helpful in making a differential diagnosis between different types of primary and secondary cardiomyopathies [11-13] (see below). In pediatric populations, cardiomyopathies are of particular significance due to the need for timely intervention to prevent morbid outcomes.

Cardiomyopathies are of different types, primary and secondary. Genetic cardiomyopathies are of the primary/instrinsic category and include: (i) Hypertrophic cardiomyopathy (HCM or HOCM), (ii) Arrhythmogenic right ventricular cardiomyopathy (ARVC), (iii) Isolated ventricular non-compaction Mitochondrial myopathy, (iv) Mixed Dilated cardiomyopathy (DCM), (v) Restrictive cardiomyopathy (RCM), (vi) Acquired Peripartum cardiomyopathy, (vii) Takotsubo cardiomyopathy and (viii) Loeffler endocarditis. Secondary/extrinsic cardiomyopathies can be categorized based on causal relationships into:

1. Metabolic/storage: Examples are: amyloidosis and hemochromatosis;

2. Inflammatory: Examples are: viral myocarditis and Chagas disease;

3. Endocrine: Examples are: diabetic cardiomyopathy, hyperthyroidism and acromegaly;

4. Toxicity: Examples are chemotherapy, and alcoholic cardiomyopathy;

5. Neuromuscular: Example: muscular dystrophy;

6. Nutritional diseases: Example: Obesity-associated cardiomyopathy; and

7. Other: "Ischemic cardiomyopathy" is a weakness in the muscle of the heart due to inadequate oxygen delivery to the myocardium with coronary artery disease being the most common cause. This aspect is not supported by current cardiomyopathies classification schemes.

Table 1 depicts statistics on pediatric populations for genetic cardiomyopathies, which include HCM [14], DCM, ARVC/D, RCM (and iron mediated CM), NCM [15], along with Tetralogy of Fallot (ToF) [16] that is a morphological congenital heart disease (CHD) associated with myopathy of the right ventricle. Some examples of cMRI-based quantitative and qualitative markers are also depicted. These biomarkers are representative of structure (morphology), function and dynamics (flow) of the heart muscle. Myocarditis is an inflammatory disease of the myocardium with a wide range of clinical presentations, from subtle to devastating [17]. We know from literature that myocarditis falls under the classification of secondary cardiomyopathies of the inflammatory subtype. The definition of myocarditis varies, but the central feature is an infection of the heart, with an inflammatory infiltrate, and damage to the heart muscle, without the blockage of coronary arteries that define a heart attack (myocardial infarction) or other common noninfectious causes. Myocarditis may or may not include death (necrosis) of myocardial tissue. It may include dilated cardiomyopathy. In this dataset collected and analyzed in this paper, we include patients who were diagnosed with myocarditis in addition to the primary genetic cardiomyopathies. Myocardial delayed enhancement (MDE) is a feature that is very often present in such patients and we were looking to see if our predictive models pick up this feature for cardiomyopathy classification from cMRI data.

**Table 1:** Incidence, prevelance and other statistics for five cardiomyopathies and a more prevalent pediatric congential heart disease called Tetralogy of Fallot or ToF, with associated right ventricular abnormalities.

CM Subtypes	HCM	DCM	ARVC/D	NCM	RCM & Iron mediated CM	ToF^16^
**Incidence (I) OR Prevalence (P)**	P = 1:500 in absence of aortic valve disease or systemic hypertension	I = 5-8 cases /100,000 P = 36 cases /100,000	I = 1/ 10,000	I = 0.05% to 0.24%	I = 11.4% to 15.1% in Thalassemia major patients Transfusion Dependent	I = 9/1000 live births

**#Patients evaluated for CM**	46	129	44	31	35	684

**Total number of positive diagnosis w/ CMRI CM**	11	18	4	15	12	119

**cMRI-based QUANTITATIVE markers**						

**LV myocardial wall thickness**	ABNL	ABNL	NL	ABNL	ABNL	NL

**LV mass index**	ABNL	ABNL	NL	ABNL	ABNL	NL

**LV Volume index**	ABNL	ABNL	NL	ABNL	ABNL	ABNL

**RV Volume index**	NL	ABNL	ABNL	NL	ABNL	ABNL

**cMRI-based QUALITATIVE markers**						

**Myocardial Delayed Enhancement**	+/-	+/-	+/-	+/-	+/-	+

**Wall motion abnormalities**	+/-	+/-	+/-	+/-	+/-	+/-

Some examples of standard quantitative and qualitative markers from cMRI that are associated with observed normal (NL) or abnormal (ABNL) values in each disease based on patients seen at the Children's Hospital of Pittsburgh (CHP) between 2000 and 2013. LV refers to Left Ventricular and RV to Right Ventricular regions. The qualitative variables shown in the table are assigned values of + or - indicating that some patients for the particular subtype indicated presence or absence of that abnormality.

Machine learning methods are now routinely applied to predictive modeling of disease states from high-dimensional biomedical data, with rule learning methods becoming useful for classification and extraction of discriminatory biomarkers [18,19]. Both linear and non-linear modeling methods are available for classification tasks, wherein a classifier is learned using training data containing possible predictors (e.g. biomarkers) of a target class (e.g. the presence or absence of a disease). A particular method that has been applied successfully to 'omic' biomarker discovery is the Bayesian rule learning (BRL) [20] system, which uses a Bayesian score to construct Bayesian networks (BNs) and to learn probabilistic rule models from them. The models produced are easily interpretable by the biomedical scientist and have been shown to have fewer markers and equivalent or greater classification performance in comparison to models derived from other rule learning methods [20,21]. In this paper, we develop and apply a novel workflow that permits the application of BRL to cMRI-derived biomarkers for classification of positive versus negative findings of cardiomyopathy in pediatric patients. The major enhancements in this journal version of our previously published conference paper [22] are: (a) we have almost doubled the amount of retrospectively obtained cMRI-derived data which led to reportable and promising cross-validation predictive performance; and (b) included a known qualitative variable (Myocardial Delayed Enhancement status) to verify predictive rules obtained from BRL.

The main hypothesis is that our novel cMRI-BED framework developed below, which includes predictive modeling of retrospectively obtained de-identified cMRI-derived biomarkers and medical record data into the current standard clinical workflows for evaluating pediatric patients for cardiomyopathy using cardiac MRI will lead to: (a) better scientific understanding of the interactions among image-derived biomarkers that impact positive or negative findings, as depicted in easy to understand IF-THEN propositional rules; and (b) provide additional statistical information to the cardiologist in terms of the prediction of positive or negative findings based on the predictive model/rules learned from training data for a new test case. The use of our Bayesian Rule Learning (BRL) methods provide a posterior probability for each rule, and since the rules are mutually exclusive, only one rule will be used for providing the prediction. These predictive IF <condition> -THEN <class> rules directly show the non-linear interactions among the various image-derived biomarkers along with statistical information which are believed to depict proof of concept for our working hypothesis.

## Methods

**Figure 1:**
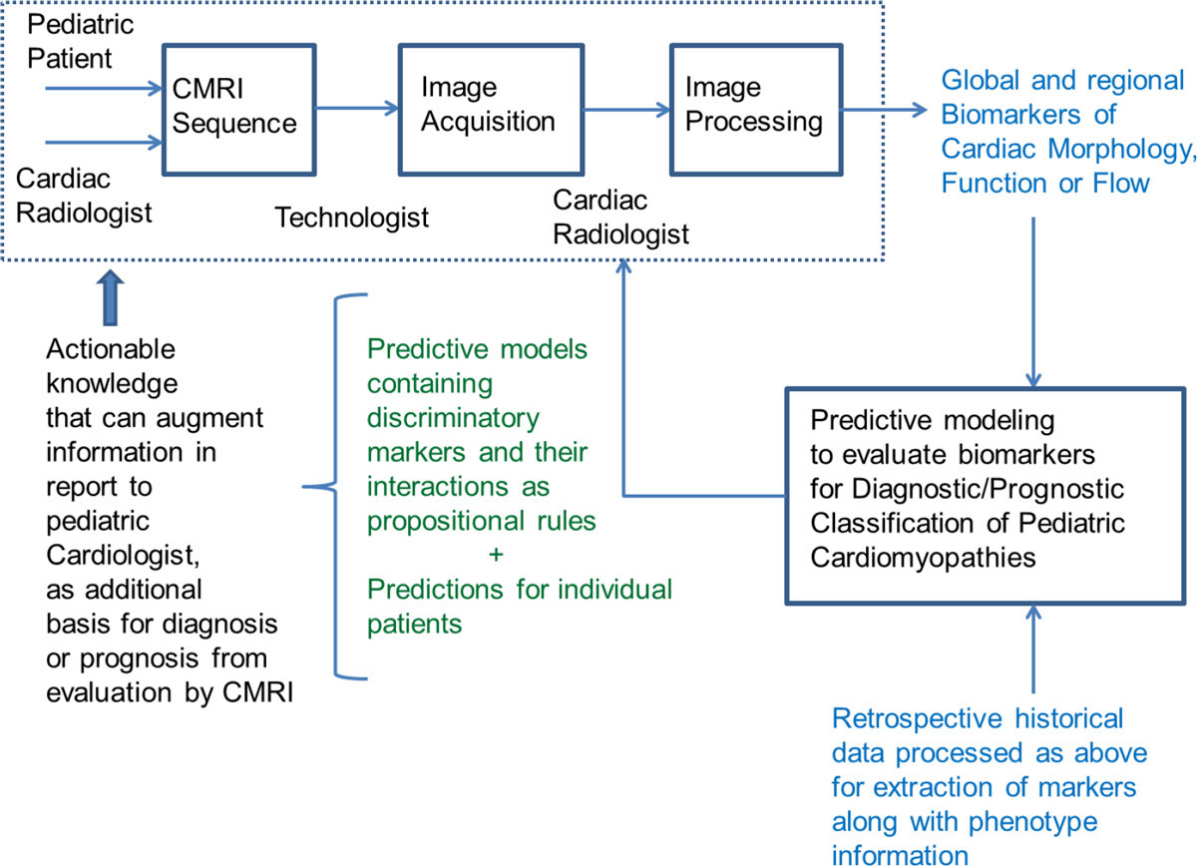
**Overview of the Cardiovascular Magnetic Resonance Imaging Biomarker Extraction and Discovery (cMRI-BED) framework**. Standard clinical practice is depicted as dotted box.

Figure 1 depicts the cMRI-BED informatics workflow which represents a simplified process description by which cMRI-derived biomarkers can be extracted and interactions among the biomarkers can be assessed using state-of-the-art predictive rule models to assist in the accurate classification of cardiomyopathies in children. The pediatric patient with a suspicion of cardiac disease based on presenting signs and symptoms is usually referred by the primary care physician (PCP) to consult with pediatric cardiology for basic initial clinical cardiac evaluation. Accurate evaluation of complicated cases necessitate advanced cardiac MRI sequences as recommended by the experienced pediatric radiologist based on initial clinical findings, family history of patient and published literature and guidelines laid down by the Society for Pediatric Radiology. These sequences dictate the preparation of the patient, and subsequent image acquisition by the technician who works together with the radiologist and technology to capture the appropriate sets of images, ensuring their quality. Phantom runs are made with a body of water placed in lieu of the patient with the same parameter settings to ensure that the values obtained by the technology are within acceptable ranges.

Once the images are acquired, which takes approximately two hours depending on the cMRI protocol, they are post-processed by the cardiac radiologist, an appropriately trained physician who can evaluate the large sets of images and mark regions and contours for biomarker quantification looking for context-dependent abnormalities. The radiologist also provides qualitative assessments for several standard markers. Commercially available image processing software technology is used to assist the radiologist in performing these assessments, and is made available through the same or other commercially available vendors at the scanning site. The commercially available software technology permits the generation of standard reports that contain quantitative and qualitative assessments of the cMRI-based diagnosis, and these reports are sent to the referring pediatric cardiologist for appropriate treatment. Within the cMRI-BED, we propose to include our novel predictive modeling tools [20] to analyze retrospective data acquired for case/control discrimination from the hospital's database for performing hypothesis driven retrospective and prospective clinical research studies (see Table 1 for availability of subjects for different CM types). We will generate classification rules that can inform the cardiac radiologist, referring pediatric cardiologist and the PCP about the kinds of interactions between different markers that can better discriminate CM subtypes for appropriate management based on a training dataset, and we will also be able to give a diagnosis/prediction for a given patient which currently does not exist in a clinical workflow model.

Using the proposed framework, we can extract both standard (see Table 2) as well as novel cMRI biomarkers [23-28] for diagnostic and prognostic purposes. An example of a novel regional imaging biomarker that was recently discovered based on our analysis of publicly available cMRIs within the Cardiac Atlas Project [29] databases, is briefly discussed next. Cardiac MRIs of 25 symptomatic patients with coronary artery disease or left ventricle impairment and 25 asymptomatic patients were used to extract cardiovascular function metrics. This also led to the discovery of a new regional imaging biomarker of cardiac function that we call RMS-P2PD [23] which calculates the root mean square (RMS) error from average phase to phase regional left ventricular endocardial displacement, and is computed on a patient specific basis. In [23], we depict how addition of this RMS-P2PD biomarker to standard biomarkers increased the leave-one-out cross validation predictive accuracy of BRL models for ischemic cardiomyopathy classification from 83.8% to 91.9%. The workflow depicted in Figure 1 is aimed to augment the efficiency and accuracy with which clinical radiologists detect and treat cardiovascular abnormalities in children.

**Table 2:** Standard cMRI biomarkers produced by ReportCARD™ system (GE Healthcare).

Left Ventricle (LV) Parameters	Right Ventricle (RV) Parameters	Overall Cardiac Parameters
A.S. Wall (cm) P.S. Wall (cm) End Diastolic Dimension (cm) End Systolic Dimension (cm) LV End Diastolic Vol (ml) LV End Systolic Vol (ml) LV Ejection Fraction (%) LV End Diastolic Index (ml/m^2^) LV End Systolic Index (ml/m^2^) Fractional Shortening (%)	RV Major Axis (cm) RV Minor Axis (cm) RV End Diastolic Vol (ml) RV End Systolic Vol (ml) RV Major Axis Index (cm/m^2^) RV Minor Axis Index (cm/m^2^) RV Ejection Fraction (%) RV End Diastolic Index (ml/m^2^) RV End Systolic Index (ml/m^2^)	Stroke Volume (ml) Stroke Volume Index (ml/m^2^) Heart Rate (bpm) Cardiac Output (l/min) Cardiac Index (l/min/m^2^)

Below we give an illustrative example for proof-of-concept of this framework, which uses available rare data from the Children's Hospital of Pittsburgh (CHP) of University of Pittsburgh Medical Center (UPMC) which has a premier heart care program. The framework can be used to assess whether or not certain types of cMRI biomarkers measured using different technologies are suitable for classification of pediatric cardiomyopathy, and if so, to what extent. An example would be to assess the value of strain quantification measures from myocardial tagging sequence using cMRI to detect the presence or absence of regional morphological changes as an early marker of cardiomyopathy [15,16] in patients referred for cardiac imaging tests. Strain quantification is a robust upcoming method for regional myocardial function evaluation, which explains the underlying pathology of cardiomyopathy that can lead to timely management via early intervention [16].

**Dataset acquisition and characteristics: **A de-identified retrospective cMRI dataset was obtained as below under an ongoing IRB approved exempt study, by collecting radiological images and electronic medical reports from the PACS and MARS servers at the UPMC. Inclusion criteria consisted of patients seen at Children's Hospital of Pittsburgh between 1998 and 2014 who received a cardiac MRI to evaluate for cardiomyopathy or myocarditis. Patients with incomplete or poor quality scans were excluded. Measurements and biomarkers, including age, sex, height, weight, BSA, ventricular volumes, masses and dimensions, wall thicknesses, myocardial delayed enhancement, heart rate, and calculated measurements thereof were extracted from the MRI reports (see Table 2 for list of standard cMRI biomarkers). The radiologist's impression was studied to determine what abnormality, if any, was present. In addition, the most recent cardiology progress note, if present, was studied to determine the cardiology diagnosis. If a patient had multiple cardiac MRIs, only the initial one was used. This minimized the time between original presenting symptoms or concern for cardiac anomaly and MRI. As a preliminary study, the dataset was filtered to eliminate patients with conflicting MRI and cardiology diagnoses or uncertain diagnoses. The resulting dataset included 83 patients age 0-22 (only four patients were between the ages of 19 and 22, with 2 males and 2 females, and equal distribution of one positive and one negative case for each gender) evaluated with cMRI at CHP for cardiomyopathy or myocarditis. For patients with multiple diagnoses (such as combined HCM and left ventricular non compaction, LVNC), a judgment was made as to which one seemed to be the primary diagnosis. Table 3 depicts the composition of the dataset including the number of patients that received a negative diagnostic finding for cardiomyopathy, and the number of patients that received a positive finding for a particular type of cardiomyopathy. The dataset contained standard cMRI biomarkers (see Table 2) along with gender, age, diagnosis and myocardial delayed enhancement (MDE) status as a binary variable. It is to be noted that a few of the markers in Table 2 such as Fractional Shortening (FS), left and right ventricular ejection fractions, cardiac output and the indices are derived parameters.

**Table 3:** Composition of patients in our retrospectively collected cMRI dataset.

MR Diagnosis	Total #patients (male, female)
HCM	9 (4, 5)

DCM	3 (1, 2)

ARVD	2 (2, 0)

LVNC	7 (4, 3)

Myocarditis	11 (9, 2)

Negative	51 (31, 20)

We constructed new variables based on the "normal" ranges for the left ventricular (LV) and right ventricular (RV) end-systolic and end-diastolic volumes and Stroke Volume parameters [30]. The parameters were normalized to the age and gender specific mean according to published control data [30] to determine whether a patient's volumes are within a normal range given his/her BSA. Patients with volumes that were two standard deviations from the mean had parameters labelled as "low" or "high", with the remaining labelled as "normal". Using this method, we created 5 new discrete variables LVEDV Range, LVESV Range, RVEDV Range, RVESV Range and Stroke Volume Range.

**Image Acquisition and Processing: **The cMRI images were acquired with a GE SignaHDxt 1.5 Tesla MRI (GE Healthcare, WI, USA). Scans were performed by highly experienced cardiac MRI technologists at CHP of UPMC. Due to their young ages, a few patients who were unable to maintain breath hold for specific cMRI sequences required general anesthesia during their MRI scans, as per their clinical protocols. Cardiac sequences for function, flow and tissue characterization analyses require 15-20 sec breath hold for image acquisition. A balanced steady state free precession sequence (FIESTA, GE) was used in the short axis to acquire images for biventricular volumetric analysis during 20 phases of the cardiac cycle. Relevant parameters included breath holds = 1-2 (none for patients under general anesthesia), number of excitations = 1 for patients with breath holds and 2 for patients under general anesthesia, repetition time = 3.6-4.0 ms, echo time = 1.5-1.7 ms, flip angle = 55°, slice thickness = 5-7 mm, and acquisition matrix = 256 ± 256. Commercially available post-processing software ReportCARDTM (GE Healthcare, WI, USA) was used to determine volumetric data, flow and velocities.

**Figure 2:**
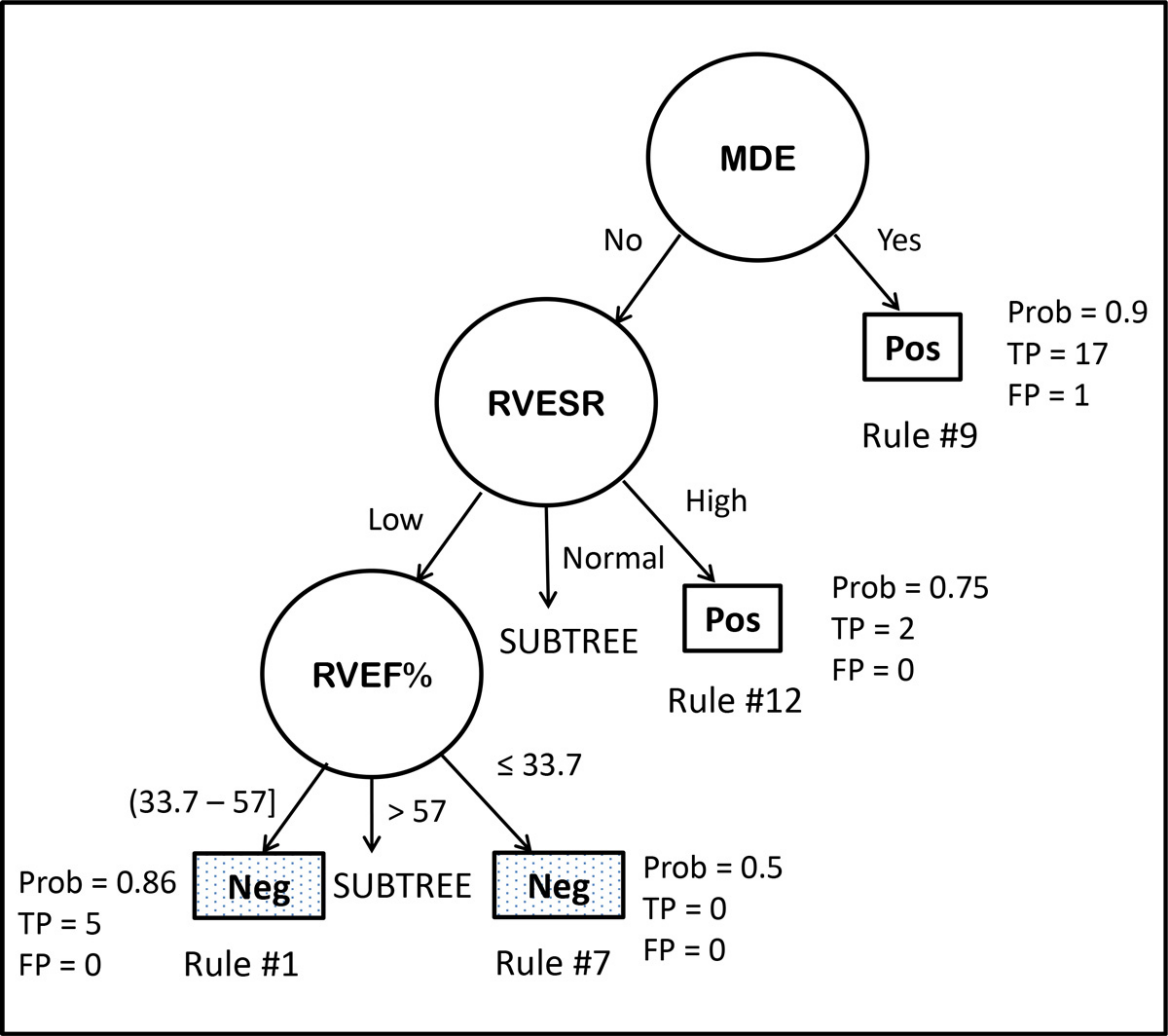
**A portion of the Bayesian Decision Tree model from BRL is depicted to visualize the classification rules**. The predictor variables are shown in circles, and their values along each branch from the root of the tree to the leaf node depicted as a rectangle, represents a classification rule within the model. The rectangular leaf node represents the target variable, MRDx value for positive (Pos) or negative (Neg) finding of cardiomyopathy. Four rules are shown with some associated statistics such as the posterior probability (Prob) of the rule, and the numbers of true positives (TP) and false positives (FP) covered by the rule. Two of the subtrees are not shown. From this tree, we can easily spot areas where evidence is weak, strong, or could be combined. The variables shown include Myocardial Delayed Enhancement (MDE), Right Ventricular End Systolic Volume Range (RVESVR), and the Right Ventricular Ejection Fraction (RVEF%).

**Data Analysis Methods: **The cMRI-derived biomarkers dataset containing 32 positive cases of cardiomyopathy/myocarditis and 51 negative controls and 30 predictor variables containing no missing data was analyzed using our novel Bayesian Rule Learning (BRL) methods [20,31]. BRL [20] works by searching for interactions between predictors that are favorable for discriminating the target class values, which for this dataset are represented by positive (Pos) or negative (Neg) MR diagnosis (MRDx). BRL performs a heuristic, iterative search of the entire space of possible models representing interactions among potential predictors, and uses a Bayesian score [20] to represent the uncertainty in the validity of each model based on the available training data. The greedy search starts with the highest scoring single predictor variable as the only parent of the target class, and in each iteration adds the next highest scoring variable as another parent to the target class and recalculates the Bayesian score [20]. The maximum number of parents or predictor variables of the target class is used to constrain the model space (default value is 8). The BRL method in [20] was extended to handle search of local structures in [31]. The Bayesian decision tree (BRL-DT) greedy search of local structures within BRL allows for inclusion of different variables and varying numbers of variables on the left hand side of related subsets of classification rules, as long as this local variation produces an improvement in the Bayesian score for the model (entire set of rules) [31]. This brief explanation is best understood by illustration of the BRL-DT model in the results section below and its tree visualization depicted in Figure 2.

BRL produces models that contain mutually exclusive and exhaustive classification rules, so that only one rule applies for prediction on a test case. When using the BRL system for developing and testing predictive models, we generally apply local structure search in addition to the original global structure search and present report results and the model from the algorithm that performed the best over cross-validation.

Apart from BRL methods, we also applied three standard machine learning methods available in the WEKA data mining environment [32]: Support Vector Machines (SVMs) [33], C4.5 decision tree learning [34] and Random Forests (an ensemble method) [35] to obtain 10-fold cross-validation performance using accuracy and area under ROC curve (AUC) measures for this dataset.

## Results

We present below a BRL decision tree [31] model to illustrate the kinds of interactions between cMRI-derived markers that can be used for automatic classification. We used a supervised discretization algorithm called Efficient Bayesian Discretization (EBD) [36] available within BRL to discretize the continuous-valued input variables. EBD requires a parameter called lambda, which is a prior that controls the total number of cut-points from the desired discretization. We empirically varied this lambda parameter between 0.5 and 4.0, in steps of 0.5 increments, and chose the lowest lambda value that resulted in best cross-validation performance. For this dataset, we set the EBD lambda parameter value to 3.5. The 10-fold cross validation accuracy we obtained on this dataset was 80.72% with an AUC of 79.6%, which gave us confidence that reasonably accurate predictive models can be obtained from cMRI-derived biomarker data. For illustrating this, we depict below the model from BRL that was obtained by learning on the entire training data (32 positives, 51 negatives). This model (see rules below) was applied to the training data to make predictions, and fit the data with the following statistics - Accuracy = 90.4%, Sensitivity = 78.13% and Specificity = 98.1% for the Positive class, and an AUC value = 90.3%. The model used 6 variables: Myocardial Delayed Enhancement (MDE), RV Ejection Fraction (RVEF%), LV End-Diastolic Volume Range (LVEDVR), LV End-Systolic Volume Range (LVESVR), RV End-Systolic Volume Range (RVESVR), and Stroke Volume Index (SVI ml/m^2 ^) as shown below (Rules #1-8 classify Neg examples and rules # 9-13 classify Pos examples):

1. IF (MDE = No) & (RVESVR = Low) & (RVEF% = (33.7 to 57]) THEN (MRDx = Neg)

Posterior Probability (Prob) = 0.857, P = 0.081, TP = 5, FP = 0

2. IF (MDE = No) & (RVESVR = Normal) & (LVEDVR = Normal) & (LVESVR = Normal) & (SVI ml/m^2 ^≤ 67) THEN (MRDx = Neg)

Prob = 0.852, P = 0.001, TP = 22, FP = 3

3. IF (MDE = No) & (RVESVR = Normal) & (LVEDVR = Low) THEN (MRDx = Neg)

Prob = 0.85, P = 0.005, TP = 16, FP = 2

4. IF (MDE = No) & (RVESVR = Normal) & (LVEDVR = Normal) & (LVESVR = High) THEN (MRDx = Neg)

Prob = 0.8, P = 0.227, TP = 3, FP = 0

5. IF (MDE = No) & (RVESVR = Low) & (RVEF% > 57) & (LVEDVR = Normal) THEN (MRDx = Neg)

Prob = 0.75, P = 0.375, TP = 2, FP = 0

6. IF (MDE = No) & (RVESVR = Low) & (RVEF% > 57) & (LVEDVR = Low) THEN (MRDx = Neg)

Prob = 0.5, P = 0.843, TP = 2, FP = 2

7. IF (MDE = No) & (RVESVR = Low) & (RVEF% ≤ 33.7) THEN (MRDx = Neg)

Prob = 0.5, P = 1.0, TP = 0, FP = 0

8. IF (MDE = No) & (RVESVR = Low) & (RVEF > 57) & (LVEDVR = High) THEN (MRDx = Neg)

Prob = 0.5, P = 1.0, TP = 0, FP = 0

9. IF (MDE = Yes) THEN (MRDx = Pos)

Prob = 0.9, P = 0.0, TP = 17, FP = 1

10. IF (MDE = No) & (RVESVR = Normal) & (LVEDVR = Normal) & (LVESVR = Low) THEN (MRDx = Pos) Prob = 0.8, P = 0.054, TP = 3, FP = 0

11. IF (MDE = No) & (RVESVR = Normal) & (LVEDVR = High) THEN (MRDx = Pos)

Prob = 0.75, P = 0.146, TP = 2, FP = 0

12. IF (MDE = No) & (RVESVR = High) THEN (MRDx = Pos)

Prob = 0.75, P = 0.146, TP = 2, FP = 0

13. IF (MDE = No) & (RVESVR = Normal) & (LVEDVR = Normal) & (LVESVR = Normal) & (SVI ml/m^2 ^> 67) THEN (MRDx = Pos)

Prob = 0.667, P = 0.386, TP = 1, FP = 0

The posterior probability for each classification rule is calculated by BRL. In addition, the rules also contain a p-value (P) that is calculated for each rule using Fisher's exact test. The number of true positives (TP) and false positives (FP) covered by each rule is also reported. This model is depicted for purposes of illustration. From the above, we notice at least a few general rules (# 2, 3 and 9) that are reasonable in terms of both accuracy and coverage. The rules obtained from BRL are mutually exclusive and exhaustive. It is interesting to note that two of the rules (#7 and 8) have no examples, and depict areas wherein there is no evidence in the data. This implies that a diagnosis of positive or negative for cardiomyopathy is equally likely given this training dataset. However, the literature may have prior information that can be used to provide evidence for one class over the other. For example, one of the features, namely right ventricular ejection fraction /RVEF% ≤ 33.7 used in rule 7 is indicative of right heart disease. By incorporating this as prior information into our Bayesian modeling framework, we would be able to change the MRDx of the rule to positive class, rather than the default majority class chosen as the diagnosis because of the lack of evidence favouring a particular class.

These and others may also indicate the need to examine the discretization cutoff ranges for discriminatory biomarkers, and also whether certain rules can be combined together to improve overall model representation. The above classification rule model is shown for illustrative purposes (see Figure 2 for a subset of the entire decision tree) to depict how a parsimonious description of a complicated cardiac biomarker dataset can be obtained using our BRL methods. The 6 variables selected by BRL are the variables that appear in the left hand side of the IF-THEN rules. They are extracted automatically by the BRL system from the rule model. Because the rule model depicted is obtained from local search of tree structures, the number of variables in each rule may vary. With the original BRL global search method [20], each rule would have all the variables, and the combination of values for the variables would vary for each rule so as to cover all the possible combinations along the left-hand side of each mutually exclusive rule for the set of rules. In the local structure tree model, we obtain a more parsimonious representation by this account.

Access to a larger training dataset will lead to more accurate classification rules and in turn, better predictions on unseen test cases which can be used for validating our models. While BRL was able to find rules that are established and well-known in the literature [30], it must be noted that different discretization methods lead to different cutoffs for the input variables. This issue must be addressed in order to enable stable models to be learned from such cMRI datasets.

In order to ensure that the data that we have collected for our study is of sufficient quality and to verify the classification performance of our method, we compared the 10-fold cross-validation accuracy and AUC measures of performance of the BRL decision tree to that of SVMs, C4.5 and Random Forests using the WEKA data mining toolkit and its default parameter settings for each method. The BRL decision tree method outperformed the other three classifiers as shown in Table 4. It is also clear from the results that this rare dataset that we have collected is of good quality for classification of positive versus negative findings of pediatric cardiomyopathy as indicated by the cross-validation performances from several popular machine learning methods using just default parameter settings and no tuning.

**Table 4:** Comparison of classifiers using 10-fold cross validation accuracy and AUC

Method	Accuracy (%)	AUC (%)
BRL-DT (EBD lambda = 3.5)	80.72	79.6

SVM (WEKA Linear kernel)	75.90	73.4

C4.5 (WEKA default settings)	78.31	73.9

Random Forests (WEKA default)	73.49	77.2

## Discussion

Preliminary results show the feasibility of our framework for processing such data while also yielding actionable predictive classification rules that can augment knowledge conveyed in cardiac radiology outcome reports. Interactions between MDE status and other cMRI parameters that are depicted in our rules warrant further investigation and validation. Predictive rules learned from cMRI data to classify positive and negative findings of cardiomyopathy can enhance scientific understanding of the underlying interactions among imaging-derived parameters.

We are proposing a framework rather than a particular method, which means that predictive modeling could be performed by other classification algorithms. We use BRL for ease of interpretability of the predictive rules, and as shown in Table 4 and in our previous studies it is comparable to or better than other popular classification algorithms and extant rule learners [20,31]. We believe that our framework is flexible enough to support (a) different predictive algorithms, and (b) novel biomarker discovery. Both aspects require a human in the loop as indicated in Figure 1. We believe that the main limitation of the methodology arises from the quality and quantities of data available for doing the predictive modeling.

cMRI cardiomyopathy data is an example of a type of BIG data that presents several informatics challenges. As seen in the results section, the collaborative efforts between cardiac radiologists, data miners, biomedical engineers, technicians and biomedical informaticians will be crucial to establish and maintain databases or electronic repositories that can be used to create knowledge for transforming patient care. Based on our experience in applying the cMRI-BED framework, we identify the following three immediate informatics challenges that require elegant state-of-the-art solutions:

1. The need for a gold-standard, secure repository for storing the cardiac MR image sequence specific manually traced contours and image annotations performed by Dr. Madan on the entire sets of 2D, 3D and 4D images apart from the raw images acquired and stored in DICOM formats for each (de-identified) patient in clinical setting. Currently, these post-processed images are pushed to PACS for clinical reporting following the post-processing at the dedicated cardiac workstation at CHP. Image retrieval of post-processed images for clinical research is a cumbersome and deliberate time exhausting task which affects the clinical research flow.

2. The need for adequately trained personnel to perform such annotations on existing CMR images. On an average, it takes at least one year to adequately train a technologist who has met prerequisites for performing clinical cardiac MRI procedure.

3. The need for a series of systematic studies that can provide adequate age, gender and clinical history matched controls that are crucial for predictive modeling of cMRI data.

Challenge #1 can be met by secure, cloud-based architectures that permit large data storage and acquisition. Filling the second need (challenge #2) can enhance the productivity of cardiac radiologists and improve clinical management. A single pediatric radiologist reads and annotates about 350 cases in a year, because each case can take anywhere from four to eight hours to capture cMRI data and process it to generate and verify reports. Meeting challenge #3 will provide power to predictive modeling studies due to availability of matched case-controls in sufficient quantities to be able to better understand and differentiate cardiac diseases. This will be necessary in order to permit translation of this methodology into clinical practice. The cross-validation accuracies and AUC performance measures reported in this paper, while promising, are still not good enough for immediate clinical use in decision making. Future work includes acquiring more data to enable accurate predictions that can used for clinical decision-making, and subsequent automation of the cMRI-BED framework to eliminate subjective bias to the extent possible.

## Conclusions

Pediatric cardiomyopathies are significant diseases that are routinely examined using cMRI. Pediatric cardiomyopathies are a heterogeneous group of serious disorders of the myocardium and are responsible for significant morbidity and mortality among children if not timely diagnosed. In this paper, we develop and test a novel workflow called cMRI-BED for biomarker extraction and discovery from cMRI data. The novelty arises from the iterative involvement and use of unique, predictive tools such as BRL to model retrospectively available cMRI data and provide physicians with knowledge that relates biomarker interactions to outcome classification. Moreover, the workflow is flexible, scalable and largely independent of technology. Advances in cMRI technology can lead to the development of new biomarkers, which can be easily incorporated into our modeling framework. Retrospective data can be obtained from multiple institutions and summarization of these using BRL will help in drawing more general conclusions. Extensions to this workflow can also be made to allow for integration of image biomarkers from multiple platforms using variants of extant algorithms for transfer learning of classification rules [37].We believe that this cMRI-BED workflow will help in the assessment of cMRI biomarkers in a timely fashion for improved diagnosis and prognosis of pediatric cardiomyopathies. The results from this feasibility study suggest that our concept is broadly applicable to the study of any cardiovascular disease using imaging-derived biomarkers, and will facilitate incorporation of computational thinking within complex clinical workflows.

## List of abbreviations used

cMRI - Cardiovascular Magnetic Resonance Imaging

CHP - Children's Hospital of Pittsburgh

UPMC - University of Pittsburgh Medical Center

CHD - Congenital Heart Disease

PACS - Picture Archiving and Communication System

MARS - Medical Archival System

BRL - Bayesian Rule Learning

IRB - Institutional Review Board

## Competing interests

The authors declare that they have no competing interests.

## Authors' contributions

VG was responsible for the initial concept development, testing of the idea, generating the results and drafting of the manuscript. PGM was involved in conceptual idea development, testing of image biomarker extraction and revision of manuscript. SM was involved in the design and development of the idea, and in contribution of clinical de-identified data for this study. SM also participated in manuscript revision and examination of predictive models. All authors have read and approved this manuscript for publication.

## Declarations

The publication of this article was supported by a grant from the National Institute of General Medical Sciences at the National Institutes of Health (NIH) in the United States of America, Award Number R01GM100387 to VG.

This article has been published as part of *BioMedical Engineering OnLine *Volume 14 Supplement 2, 2015: Select articles from the 2nd International Work-Conference on Bioinformatics and Biomedical Engineering (IWBBIO 2014). The full contents of the supplement are available online at http://www.biomedical-engineering-online.com/supplements/14/S2.
